# Spatiotemporal patterns of malaria at cross-boundaries area in Menoreh Hills, Java, Indonesia

**DOI:** 10.1186/s12936-019-2717-y

**Published:** 2019-03-15

**Authors:** Dwi Sarwani Sri Rejeki, Anis Fuad, Barandi Sapta Widartono, E. Elsa Herdiana Murhandarwati, Hari Kusnanto

**Affiliations:** 1 Department of Public Health, Faculty of Health Sciences, Universitas Jenderal Soedirman, Purwokerto, Indonesia; 2 Graduate Program of Public Health, Faculty of Medicine, Public Health and Nursing, Universitas Gadjah Mada, Yogyakarta, Indonesia; 3 Cartography and Remote Sensing Study Program, Department of Geographic Information Science, Faculty of Geography, Universitas Gadjah Mada, Yogyakarta, Indonesia; 4 Department of Parasitology and Center for Tropical Medicine, Faculty of Medicine, Public Health and Nursing, Universitas Gadjah Mada, Yogyakarta, Indonesia

**Keywords:** Malaria, Spatiotemporal, Districts cross-boundaries, Menoreh Hills, Java

## Abstract

**Background:**

Comprehensive reports of malaria in Menoreh Hills, Central Java, Indonesia, a unique district cross-boundaries area under three districts and two provinces have been published previously. However, no study was performed to identify the hotspots of malaria in this cross-boundaries area, Kaligesing and Bagelen Subdistricts in Purworejo, Jawa Tengah Province and Kokap Subdistrict in Kulon Progo, Yogyakarta Province, using a longitudinal spatial data.

**Methods:**

Monthly reports of malaria cases at primary health centres during 2005–2015 were collected and processed with ArcGIS and SaTScan to identify the malaria distribution at the village level. Malaria distribution was analysed using global spatial autocorrelation (Moran index) in ArcGIS. Cluster analysis was conducted using SaTScan purely spatial clustering and purely temporal clustering. Cluster characteristics resulted from three different approach were compared and analysed.

**Results:**

During the last 11 years, 3812 malaria cases were reported and the number of high case incidence (HCI) villages were increased continuously. Malaria spatial distribution in Menoreh Hills was clustered spatially. Using three different approaches of time period ranges, consistent conclusion was found i.e. most likely clusters always occurred in the Purworejo district while the secondary clusters appeared later in the cross-boundaries districts.

**Conclusion:**

Spatiotemporal analysis of an 11 years surveillance data showed that hotspots of malaria cases in Menoreh Hills were continuously located in Purworejo district. The success of malaria elimination in the cross boundaries area of Menoreh Hills might be depended on the success in malaria case management and surveillance in this hotspot area.

## Background

Although annual parasite incidence (API) of malaria in Java has decreased, some pocket areas still exist and remain difficult to control. One of them is Menoreh Hills that belongs under three districts (Purworejo, Magelang and Kulon Progo) of two provinces (Jawa Tengah and Yogyakarta). Malaria is persistently endemic in this cross-boundaries area. In the last decade, malaria in Menoreh Hills was first documented as endemic and in fact, was the largest contributor of malaria cases in Java [1]. Malaria problems across the borders of Menoreh Hills involve several different districts and provinces, each having different strategies to control malaria. Malaria across borders or cross-border malaria is defined by specific environmental characteristics including physical, social and geopolitical, anthropological, administrative and geographic areas of border areas that influence epidemiology and control of malaria. The term cross-border malaria refers to the transmission of malaria as a result of human or vector cross-border movement and epidemiology pertaining to adjacent areas [2], a situation that is similar to the cross-border areas in Mekong [3], China–Myanmar [4], China and 14 neighbouring countries [5], Cambodia [6], and Indonesia–Malaysia [7]. The concentration of malaria events along international borders and cross-border migration activities make monitoring and control of malaria extremely difficult. Lack of monitoring and consistent surveillance can contribute to the causes of reoccurring malaria transmission.

The Menoreh Hills area consists of mainly Kulon Progo and Purworejo Districts that belong to Yogyakarta and Jawa Tengah Provinces, respectively. In Kulon Progo, malaria incidences were found in Kokap, Girimulyo, Samigaluh and Kalibawang sub-districts located in Menoreh Hills. The Kokap region is an area with dense vegetation, where temperature and humidity do not fluctuate sharply, providing a suitable environment for mosquito breeding areas [8, 9]. Thus, although API tended to decline from 2005 to 2010, outbreaks still occurred in the next period of 2011–2012 at several villages [8].

Purworejo District was the largest contributor to malaria cases in Jawa Tengah Province with API number 0.49 per 1000 population in 2010, which increased to 1.31 per 1000 population in 2011. Malaria cases in Purworejo occurred around the hills, border areas, rivers, and paddy fields [10]. Malaria outbreaks were concentrated in areas adjacent to Magelang and Kulon Progo districts. The malaria endemic areas in Purworejo, i.e., Bagelen and Kaligesing Subdistricts, are adjacent to Kokap, Kulon Progo. Throughout the year, the Anopheles species accounts for transmission in 76–88% of malaria endemic areas in Purworejo, with vector density of 0.38–3.85 mosquitoes/individuals/hour [11].

This cross-boundaries malaria situation in Menoreh Hills is similar to cross-boundary malaria transmission between countries, which is often very difficult to control. One of the reasons is that it involves different countries which apply different policies regarding malaria control. Although the three districts/two provinces in Menoreh Hills are in the same country, as decentralization era was starting in Indonesia since 2000, each district has autonomous authority to enact their own policies including those for malaria control. As a consequence, administrative issues might influence policy execution and often become a hindrance in malaria control [8].

Malaria transmission in Menoreh Hills and its surroundings varies depending on the time and place [1, 8, 10]. Information about transmission is indispensable to formulate an appropriate strategy to control malaria. Geospatial analysis is currently being developed to determine the target of intervention based on local transmission. Spatial, temporal and spatiotemporal epidemiology provide information about spatial patterns of malaria epidemics, assessing transmission changes and identifying high-risk areas and times [12–15]. In some countries such as Ethiopia, Bangladesh, China and Kenya, distribution of malaria incidence was clustered into spatial clusters of both, most likely and secondary clusters [13, 16–18] and clustered at specific times [14, 19]. Several studies on the usage of spatial and temporal information in malaria surveillance at district levels were conducted in Hubei Province of China [19], Ethiopia [16], Thailand [20] and Ethiopian highlands [21]. Studies in Cambodia [22], Ethiopia [14] and Nepal [23] used a spatial analysis unit at the village level. Spatiotemporal clustering of malaria in the villages is indispensable for government collaboration, especially for malaria control and prevention partners to provide appropriate malaria interventions and resources allocation [14]. The spatiotemporal analysis in epidemiology is extremely useful, particularly to evaluate the occurrence of different events according to the geographic areas and to identify the patterns of clustering of disease. The result of spatiotemporal mapping can be used as a tool in health policy, decision-making, and implementation of activities related to malaria elimination [24, 25]. So far, unfortunately, no spatial analysis is available in the Menoreh Hills region especially those at cross-boundaries area.

The objective of the study was to investigate spatial and temporal distribution of malaria at Menoreh Hills area, with three different approaches of analysis and to identify the challenges and solutions of the malaria control programmes in the bordering areas of Menoreh Hills.

## Methods

### Study area

**Fig. 1:**
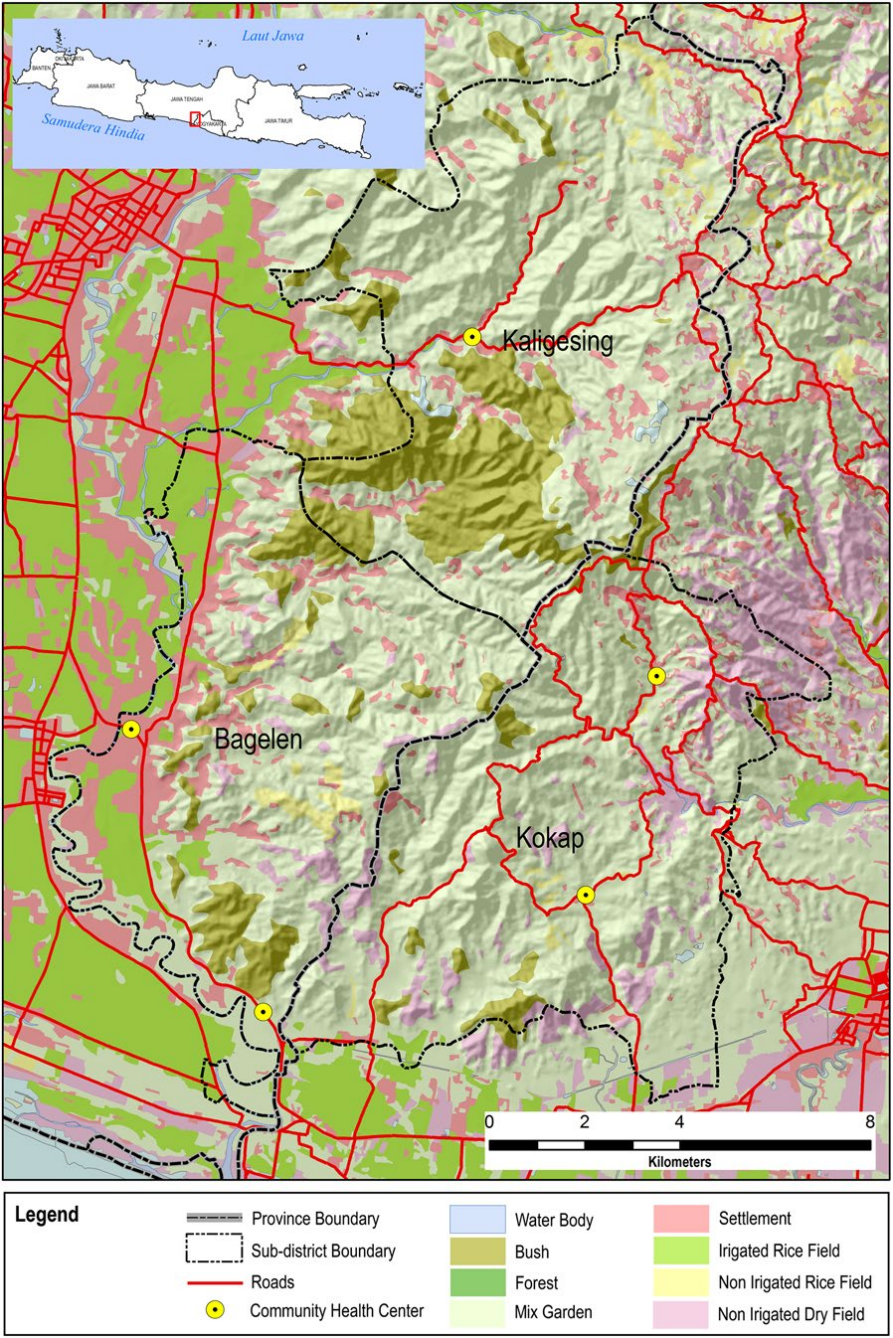
Menoreh Hill is located at cross-boundaries area of two provinces (Central Java and Yogyakarta) on the Island of Java (red box, inset), Indonesia. The topography of Menoreh Hill (Kaligesing, Bagelen and Kokap Sub Districts) consists of hilly reliefs and ephemeral water bodies (rivers/streams). Most of land area is covered by mixed gardens and shrubs, fertile in the rainy season. Puddles are presence in the rivers at the beginning and end of the rainy seasons

The study was conducted across the boundaries of districts and provinces in the Menoreh Hills of Java Island Indonesia (Fig. 1). Menoreh Hills is an area with an altitude of 100–900 m above sea level; hardy plants species are dominant in this ecosystem. Menoreh consists of many valleys and mountains that form many streams dominated by denudational mountain and hills with the dominant type of rock is andesite [8].

Purworejo district consists of 16 sub-districts and 434 villages, while Kulon Progo district consists of 12 sub-districts and 88 villages. There were three endemic areas with malaria cases always present in the last 10 years, adjacent to each other in this study: Bagelen and Kaligesing subdistricts in Purworejo district and Kokap subdistrict in Kulon Progo district. Endemicity is categorized based on the rates of API as low (API < 1.0), middle (API is between 1.0 and 4.9) and high (API ≥ 5.0) per mil population (‰).

The study area covered 43 villages. The population in the three sub-districts is 104,595, i.e., 8.7% of the total population in Purworejo and Kulon Progo.

### Data collection

Confirmed malaria cases (microscopic assessment) monthly report data were collected retrospectively from the Primary Health Care offices (PHCs) from January 2005 to December 2015. This data contained the identity of patients (including address), and malaria cases confirmed through microscopic examination. Monthly malaria data per village and the number of villagers per year were collected from related PHC a recapitulated at the district level. Village coordinate data included the coordinates of centroid latitude and longitude of each village in Menoreh Hills.

### Analysis

Data analysis included: (1) Spatial Autocorrelation Analysis (Moran Index), (2) Spatial cluster analysis and (3) Temporal analysis. Spatial autocorrelation analysis was used to analyse the patterns of malaria distribution in all villages in Menoreh Hills each year by generating Moran global indices. The determination of the patterns of distribution was determined by Z value and the level of significance (*p*-value). This analysis produced three patterns: cluster, random (no autocorrelation) and dispersed with software ArcGIS 10.2. Spatial cluster analysis, using purely spatial clustering in SaTScan software version 8.0 [26]. The temporal analysis was used to detect malaria grouping based on time of each year from 2005 to 2015 with a time aggregation 1 month. All data were analysed in three approaches, i.e., in one whole period, 11 years (2005–2015), two periods (2005–2010 and 2011–2015).

## Results

**Fig. 2:**
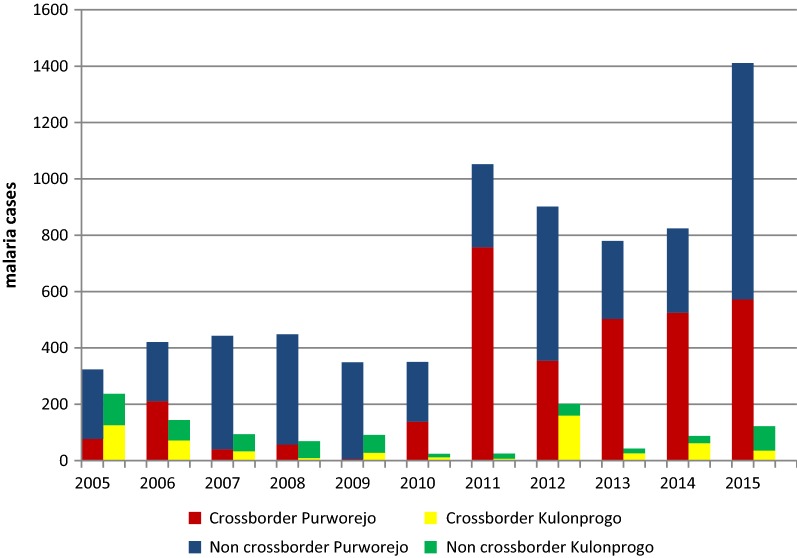
Distribution of malaria cases in cross border and non cross border areas in Menoreh Hills, Indonesia (2005–2015)

Malaria has been a long-standing health problem across the boundaries of Menoreh Hills. Over the past 11 years, more than 3500 cases of malaria in District cross-boundary area have been reported. The following were characteristics of malaria patients across the border in the Menoreh Hills: infants 0.7% (29/3812), 1–5 years old 7.3% (282/3812), ages over 5 years old 92.9% (3501/3812) with *Plasmodium falciparum* 58.5%, *Plasmodium vivax* 28.9%, mixed of *P. falciparum* and *P. vivax* 3.5% and unidentified species was 9.1%. During the study period, the API fluctuated with average at 4.2‰ and varied from 0.2–10.8 ‰. Distribution of malaria in cross-border and non-cross border areas in two adjacent districts are shown in Fig. 2.

It was revealed that Menoreh cross-border areas were the highest contributor of malaria cases in the two regencies (39–47% during 2011–2015). In the last 5 years, malaria cases in the cross-border areas of Purworejo were increasing significantly, while those on the Kulon Progo side were decreasing persistently. In the study location, the number of malaria cases reached 809 in 2005–2010 and increased significantly to 3003 in 2011–2015.

**Fig. 3:**
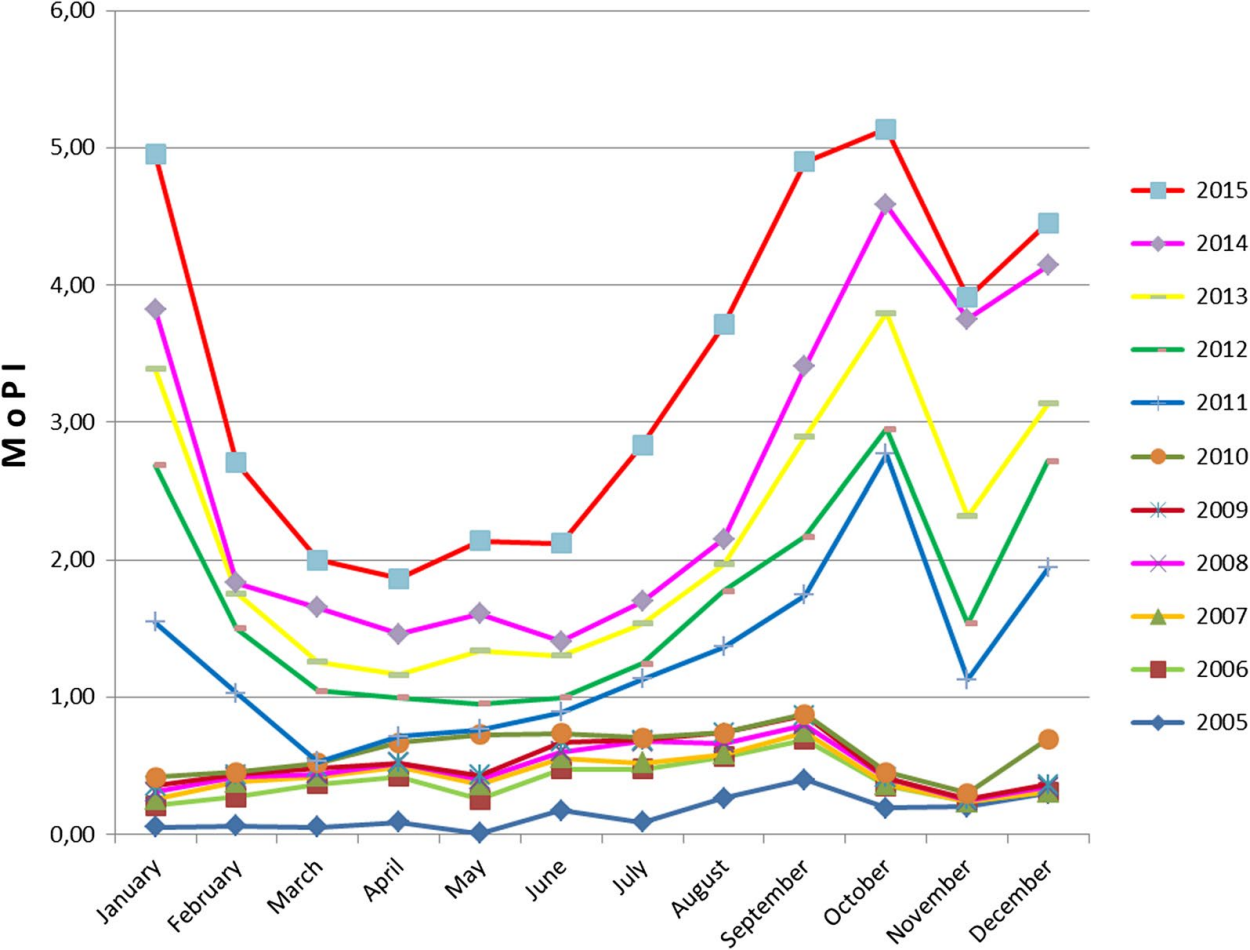
Monthly Parasite Incidence (MoPI) in cross-boundaries area, Menoreh Hills, Indonesia (2005–2015)

Monthly Parasite Incidence (MoPI) data in cross-border areas for 11 years collected from related District Health Offices showed that MoPI increased from July to January (rainy season) and decreased from February to June (dry season). MoPI value from 2005 to 2015 showed an increase with a peak value in 2015 (Fig. 3) MoPI in the last 5 years (2011–2015) showed the same pattern.

Endemicity of malaria in villages in Menoreh Hills varied for 11 years. In the last 5 years, the number of endemic villages increased, as did Low (LCI), Middle Case Incidence (MCI), and HCI villages. The number of HCI villages increased from 11 (25.6%) in 2014 to 17 (39.5%) and eventually each district in Menoreh Hills had HCI villages in 2015.

### Global spatial autocorrelation analysis (Moran Index)

Eleven years analysis showed that 42 villages reported malaria cases and a clustering pattern of malaria distribution. Two periods of analysis showed that villages with malaria cases increased from 37 in 2005–2010 to 40 in 2010–2015. Moran global index showed that there were clustering patterns in 2005–2010 that changed to random pattern/no autocorrelation in 2011–2015. Annual analysis showed that the majority of malaria cases were clustered. The number of villages reporting malaria cases in 2005–2015 ranged from approximately 9 to 32 villages, and there was an escalating trend in the last 5 years (Table 1).

**Table 1:** Incidence of malaria and its global spatial autocorrelation in the cross-border area of Menoreh Hills, Indonesia 2005–2015

Years	N	Incidence Rate	I	Z	*p*-value	
11 years (2005–2015)						
2005–2015	42		0.088	32.74	0.00	Clustered
2 periods (2005–2010 and 2011–2015)						
2005–2010	37	1.3	0.049	6.27	0.00	Clustered
2011–2015	40	7.7	0.007	1.10	0.26	Random
All years						
2005	24	1.3	0.01	8.12	0.01	Clustered
2006	29	3.0	0.09	2.29	0.05	Clustered
2007	13	0.6	0.03	6.16	0.01	Clustered
2008	15	0.9	− 0.04	− 0.44	0	Random
2009	9	0.2	0.04	4.8	0.01	Clustered
2010	17	1.8	0.02	5.91	0.01	Clustered
2011	32	9.3	0,1	5.47	0.01	Clustered
2012	30	5.9	− 0.01	0	0	Random
2013	32	6.1	0.06	3.71	0.01	Clustered
2014	27	6.2	0.01	1.11	0	Random
2015	32	10.8	0.1	5.1	0.01	Clustered

*N* the number of village reporting malaria cases, *I* the global Moran’s coefficient, *Z* the global Moran’s statistic value

### Spatial clustering of Malaria during 2005–2015

Spatial clustering method was performed using SaTScan software to detect high-risk clustering. Long-term analysis result for 11 years showed that eight villages in Purworejo District were classified into the category of *most likely clusters*. Two periods analysis showed that there were more villages in most likely cluster category during 2005–2010 (24 villages) compared to 2011–2015 (15 villages). Although less in number, the radius of the cluster was shorter in the 2011–2015 period, indicating a serious malaria problem in this area. The annual result showed that the number of most likely cluster villages varied between 2 and 15 villages. The majority of most likely cluster areas (during 2005–2015) were located in Purworejo District (highest amount of annual frequency was found in Jatirejo Village, i.e., nine times) meanwhile villages in Kulon Progo District were detected into most likely cluster once in 2005 (Table 2).

**Table 2:** The *clusters* of malaria cases detected using the *purely spatial clustering* in cross-border area of Menoreh Hills, Indonesia 2005–2015

Years	Type	N	Coordinates/radius	Observed	Expected	RR	LLR	*p*-value
11 years (2005–2015)								
2005–2015	A	8	− 7.761 S, 110.035 E/6.42 km	2573	1068.15	4.39	1164.28	0.000
	B	5	− 7.796 S, 110.079 E/1.83 km	667	195.77	3.84	373.86	0.000
2 periods (2005–2010 and 2011–2015)								
2005–2010	A	24	− 7.691 S, 110.012 E/15.2 km	461	212.67	3.74	169.8	0.000
	B	11	− 7.691 S, 110.091 E/4.02 km	239	71.10	4.36	142.76	0.000
2011–2015	A	15	− 7.761 S, 110.035 E/6.42 km	2414	1424.23	3.11	554.70	0.000
	B	3	− 7.796 S, 110.079 E/2.32 km	1064	543.66	2.36	241.21	0.000
All years								
2005	A	9	− 7.691 S, 110.012 E/15.27 km	461	212.7	3.74	169.84	0.000
	B	1	− 7.819 S, 110.115 E/0 km	23	1.18	21.87	47.76	0.000
2006	A	15	− 7.731 S, 110.088 E/5.67 km	160	81.47	3.07	45.66	0.000
	B	5	− 7.696 S, 110.103 E/1.95 km	69	18.71	4.49	44.54	0.000
2007	A	6	− 7.691 S, 110.091 E/3.60 km	17	2.97	7.35	17.30	0.000
2008	A	6	− 7.696 S, 110.103 E/2.34 km	51	21.68	2.35	6.95	0.000
2009	A	5	− 7.691 S, 110.091 E/11.21 km	7	2.07	3.86	3.91	0.030
2010	A	10	− 7.696 S, 110.103 E/11.19 km	99	27.02	15.13	95.93	0.000
	B	2	− 7.789 S, 110.064 E/1.93 km	32	1.36	31.47	74.62	0.000
2011	A	13	− 7.761 S, 110.035 E/6.42 km	704	298.73	6.72	374.09	0.000
	B	5	− 7.789 S, 110.064 E/2.73 km	385	159.01	3.44	151.77	0.000
2012	A	2	− 7.713 S, 110.062 E/1.43 km	73	13.88	5.91	65.42	0.000
	B	9	− 7.776 S, 110,085 E/4.58 km	285	196.94	192	29.15	0.000
2013	A	13	− 7.780 S, 110,049 E/5.25 km	419	237.44	3.83	117.78	0.000
	B	2	− 7.780 S, 110.047 E/1.62 km	120	47.45	2.53	44.03	0.000
2014	A	13	− 7.780 S, 110.049 E/5.16 km	414	230.21	3.69	117.32	0.000
	B	1	− 7.776 S, 110.085 E/0 km	124	47.99	3.01	47.31	0.000
2015	A	2	− 7.796 S, 110.079 E/1.83 km	247	71.74	3.44	148.75	0.000
	B	10	− 7.713 S, 110.062 E/5.75 km	373	294.21	1.44	14.60	0.000

*Type* A: type of most likely cluster and B: second most likely cluster, *N* the cluster number of village was identified by Kulldorff’s spatial scan, *RR* relative risk, *LL* log likelihood ratio

**Fig. 4:**
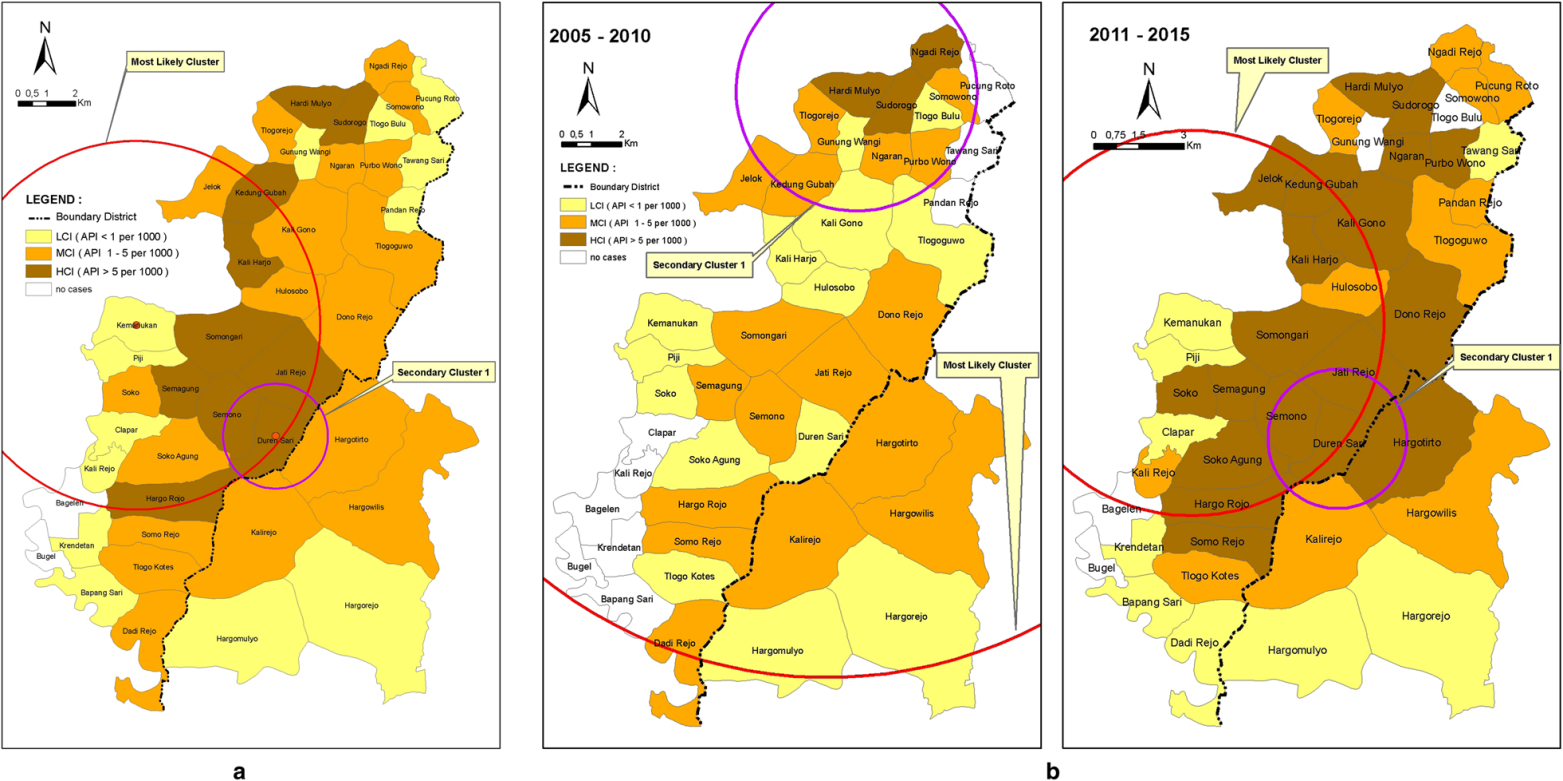
Overlay *of spatial clusters of* malaria cases in cross-boundaries Menoreh Hills, Indonesia, identified from 2005 to 2015 analyses: **a** as a whole period (11 years), and **b** in 2 time frame periods (2005–2010 and 2011–2015). Most likely clusters are presented by red circles and secondary clusters by purple circle

**Fig. 5:**
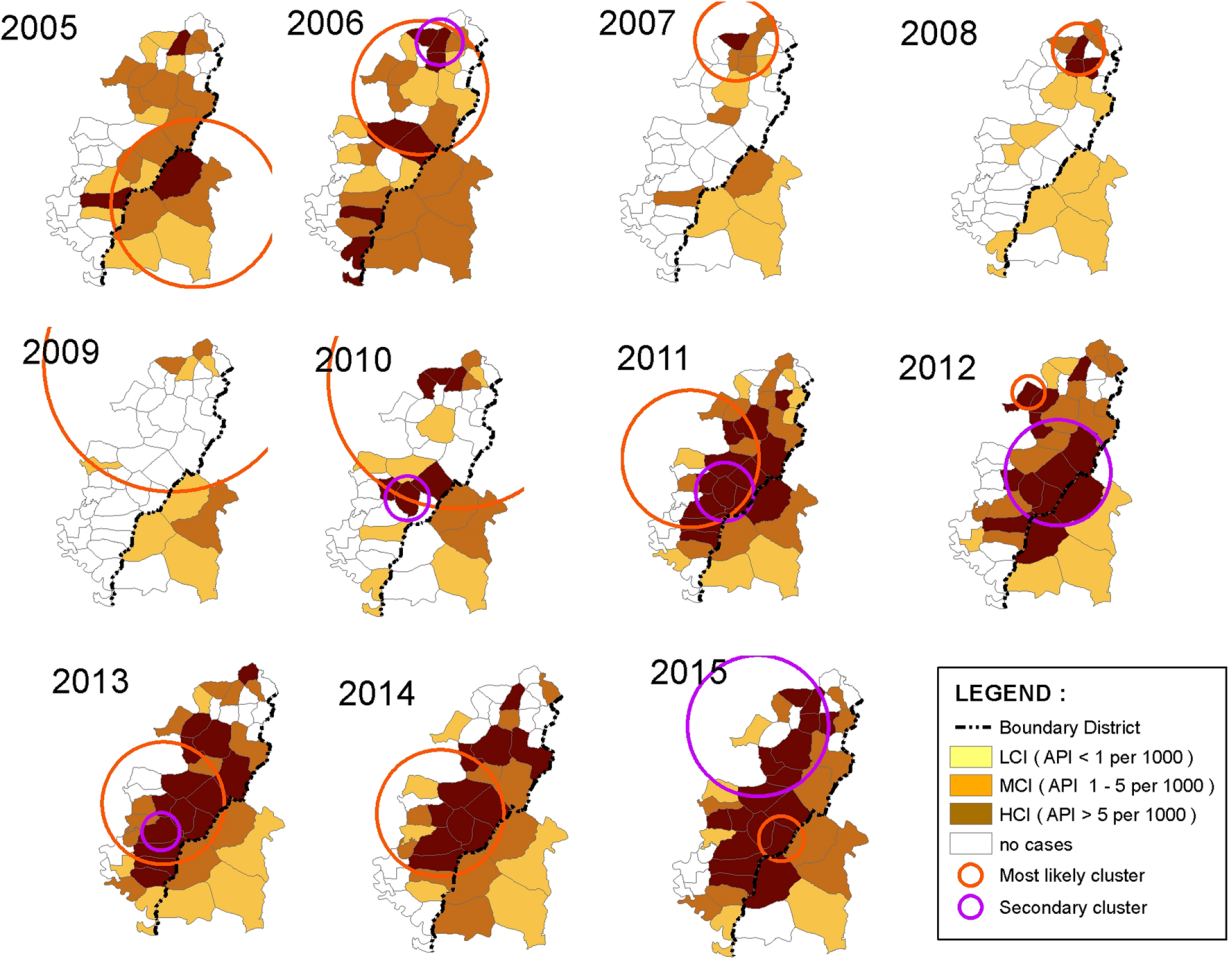
Overlay of *spatial clusters of* malaria cases identified from 2005 to 2015 in cross-boundaries Menoreh Hills, Indonesia, analysed annually. Most likely clusters are presented by red circles and secondary clusters by purple circles

Figure 4 shows the most likely clusters and secondary clusters from 11 years and two periods (2005–2010 and 2011–2015) analysis in Menoreh. Eleven (11) years analysis concluded that the target of malaria problem in cross-boundary Menoreh Hills (the most likely cluster) was Purworejo District, which was classified as an HCI and MCI area. Meanwhile, two periods of analysis showed that malaria cases were evenly distributed amongst the three sub-districts of both regencies during 2005–2010, demonstrated by the most likely cluster with 15.2 km radius and secondary cluster in Kaligesing sub-district. In the second period (2011–2015), the most likely cluster was still in Purworejo District, although it was shifted with only a 6.42 km radius, centred in Kemanukan Village, Bagelen sub-district, and was dominated by HCI villages. The secondary cluster area in 2011–2015 was a cross-border area in the two regencies. The villages in this area were classified into HCI and MCI categories. Annual analysis result (Fig. 5) showed that the category of most likely cluster frequently occurred in Purworejo. Most likely cluster patterning happened in Kulon Progo twice in 2005 and 2013 in the cross-boundaries districts. These most likely cluster areas were dominated by villages with HCI and MCI categories.

### Distribution of malaria temporal clustering

Temporal cluster analysis was used to determine the duration of clustering in the cross-border areas of Menoreh Hills. Table 3 is the result of temporal analysis of malaria cases with three approaches of analysis (11 years, two periods, and annual analysis) using SaTScan with purely spatial analysis. Temporal cluster analysis showed clustering of malaria cases occurred for 15 months (1 August 2005 to 30 October 2006) in 2005–2010 and 16 months (1 September 2011 to 31 December 2012 in 2010–2015) with average cases per month of 45 and 30 cases, respectively.

**Table 3:** The *clusters* of malaria cases detected using the *purely temporal clustering* in cross-border area of Menoreh Hills, Indonesia 2005–2015

Years	Cluster time frame	Observed	Expected	RR	LLR	*p*-value
11 years (2005–2015)						
2005–2015	1/12/2010–31/10/2015	3586	1967.24	5.45	1261.8	0.001
2 periods (2005–2010 and 2011–2015)						
2005–2010	1/08/2005–31/10/2006	444	167.70	4.68	227.26	0.001
2011–2015	1/09/2011–31/12/2012	714	301.31	2.71	230.31	0.001
All years						
2005	1/08/2005–31/12/2005	149	87.19	3.50	37.56	0.001
2006	1/03/2006–31/8/2006	205	151.23	2.12	19.75	0.001
2007	1/02/2007–30/6/2007	47	27.12	3.55	12.23	0.001
2008	1/07/2008–31/8/2008	26	11.18	3.19	9.33	0.001
2009	1/06/2009–30/09/2009	24	14.04	2.66	4.94	0.016
2010	1/12/2010–31/12/2010	38	10.36	4.87	25.49	0.001
2011	1/09/2011–31/12/2011	588	305.51	3.50	176.01	0.001
2012	1/01/2012–31/01/2012	126	46.92	3.18	51.89	0.001
2013	1/09/2013–30/11/2013	256	143.11	2.42	52.27	0.001
2014	1/10/2014–31/12/2014	336	148.46	3.94	134.13	0.001
2015	1/07/2015–30/09/2015	435	240.96	2.48	92.01	0.001

*RR* relative risk, *LLR* log likelihood ratio

Temporal cluster analysis in the 11 years showed a longer duration of clustering, including 59 months with an average of 61 cases per month. Annual temporal cluster occurred in different months as shown in Table 4. The average number of clustered malaria cases per month from 2005 to 2015 were 30, 35, 10, 13, 6, 38, 147, 126, 86, 112 and 152, respectively. The summary of annual spatial and temporal analysis approaches are shown in Table 4.

**Table 4:** Annual spatial and temporal analysis in the whole 11 years, 2 periods (2005–2010 and 2010–2015) and annually, in Menoreh Hills, Indonesia

	11 years (2005–2015)	2 periods (2005–2010 and 2011–2015)	All years
Annual spatial using SaTScan	Primary clustering occurred in Purworejo and secondary occurred in cross-border area of both regencies	Primary clustering of malaria in 2005–2010 occurred in both regencies. However, in 2011–2015, more clustering occurred in Purworejo	In general, clustering occurred more often in Purworejo district. In 2009–2014, both primary or secondary cluster occurred more often in Purworejo. In 2015, clustering occurred in the cross-border area
Temporal cluster	Cluster duration during the 11 years was 59 months with a case average clustering of 61 cases per month	Cluster duration during 2005–2010 was 15 months with an average of 30 cases per month including 16 months with an average of 45 cases per month during 2011–2015	Cluster duration from 2005 to 2015 was different in each month. Average of cases per month in cluster varied. The lowest was 6 cases in 2009 and the highest was 152 cases per month in 2015. Starting from 2011, clustering average in study location increased

## Discussion

API in Menoreh Hills fluctuated during the last 11 years. In general, API was decreasing from 2005 to 2009, and then was increasing from 2011 to 2015. The number of HCI villages increased from 2005 to 2015. In 2015, the villages in Menoreh Hills were dominated by HCI category. All analyses from the three approaches [long term (11 years), two (2) periods (2005–2010 and 2011–2015), and annual analysis] showed a clustering of malaria cases in cross-border areas of Menoreh Hills. Long-term analysis showed clustering patterns, while two periods of analysis showed random patterns, especially during 2008 and 2012. Spatiotemporal analysis has been commonly used to identify clustering or non-clustering patterns and the locations e.g., a study in Hubei Province, China, which showed non-random purely spatial cluster in 2004–2011 and identified 11 regencies as malaria high risk locations [19], a study in Ethiopia that showed clustering pattern among outpatients diagnosed with malaria and identified consistent location every year [21], and a study in Peruvia that identified temporal clustering which occurred in April-June [27].

ArcGis analysis only analysed the coordinates of clustering distribution while SaTScan analysed both coordinates and its proximity time, thus autocorrelation using ArcGIS was unable to determine cluster location while SaTScan analysis could provide information about cluster radius, allowing comparison between disease risk inside and outside the cluster [23]. Using three different approaches for analysis, the differences and benefits of spatial analysis in a long-term time period can be shown. The benefits of the three approaches of retrospective analysis are to obtain a general idea about the heterogenicity of malaria in the Menoreh Hills region and locate the specific malaria clustering sites. Eleven years of analysis showed the Menoreh Hills region as a whole was endemic for malaria. Two periods of analysis showed that the most endemic areas were mostly located inside the Purworejo regency, whereas annual analysis showed specifically that malaria cases were located in villages inside the Menoreh Hills region. The trend of malaria endemicity can be seen specifically in the yearly analysis.

The three approaches analysis using SaTScan showed that the main target of the malaria problem in cross-border areas of Menoreh Hills (the most likely cluster) was the Purworejo District, while secondary clusters might involve both districts. The areas of most likely clusters and secondary clusters were villages with HCI and MCI categories. There were villages that were always involved in primary clustering, that is villages in Purworejo District whereas villages in Kokap sub-district, Kulon Progo District were only secondary cluster villages. Based on the data, the main problem of malaria was concluded to be in the cross-border areas of Menoreh Hills, specifically in Kaligesing and Bagelen Sub-districts in Purworejo, resulting in a cross-border transmission towards Kokap Sub-district, Kulon Progo. SaTScan result for long-term (11 years) analysis identified two significant spatial clusters during 2005–2015, while two periods analysis identified two significant spatial clusters in both 2005–2010 and 2011–2015 periods. The annual analysis identified 19 significant spatial clusters. Analysis of spatiotemporal studies is often used to conduct an intervention particularly to target villages and cities [28], within cross-border areas with high incidence of malaria such as Greater Mekong Subregion (GMS) [3, 29]. Strategies undertaken on the China–Myanmar border, which simplified the processes and shown interventions in cross-border areas can reduce about 90% of the burden of malaria [30]. Although the results show consistency in term of the source of transmission, some limitations related to the data sources, data aggregation and spatial analysis are acknowledged in this study. In terms of data sources, data was collected from the primary health centres (PHC) including the address of any malaria cases. According to the malaria surveillance SOP, surveillance officer in PHC should conduct a home visit for epidemiological investigation based on the address location. Since no sampling check was done to ensure data quality, it could be a limitation of the study.

Due to the retrospective approach, visiting all cases and collecting geo-coordinate of malaria cases at home was not possible. Therefore, in this study, cases were aggregated by the village. Satscan allows for spatial analysis by area (thematic map), even though the accuracy was less accurate than the individual coordinate. However, previous papers in Malaria Journal also performed spatiotemporal analysis using thematic map [18, 19]. Satscan Spatial method allows for identification of cluster either in circular or ellipsoidal form. However, this study considers only the circular form cluster. Since mostly all villages involved in this study had square form (90%) rather than rectangle shape, we are quite a confidence with the results.

Purely temporal cluster analysis was used to show the duration of malaria risk. From the three approaches of analysis, it is shown that long term analysis produced longer duration of malaria clustering compared to annual analysis. The annual analysis is used to determine which month in a year is involved in the malaria cluster. No consistent pattern was found from the result of purely temporal cluster analysis in Menoreh Hills for 11 years. Cluster duration was different every year. However, malaria clustering generally occurred more often in July–January. This clustering pattern was similar to the well-documented monthly malaria case incidence pattern, which increased from July–January then gradually decreased. This situation leads to a supported recommendation to do malaria intervention before July in the adjacent areas.

Aside from the receptive status area [1, 28, 31–33], the high cross-boundary mobility in Menoreh Hills also influences malaria transmission [8–10]. Many of the local people visit each other for trading, working, socializing, religious events and recreation; however, the migration surveillance was not sufficient, resulting in unabated malaria transmission in this region. Malaria control efforts of the two regencies were not integrated in the cross-boundary region, resulting in the persistent difficulty to eliminate malaria in the Menoreh Hills. Lesson learned from cross-border malaria studies in China–Great Mekong [3, 29, 34, 35], China–Vietnam [30], MOSASWA (Mozambique, South Africa, and Swaziland) [36], China–Myanmar [30, 37] demonstrated how important joint efforts between countries in the cross-border area are needed to control malaria. A cross-boundary fast response team is also needed to limit the period of transmission. Ignoring primary clustering in other districts which are actually in the same ecosystem area, cannot help malaria elimination efforts being pursued by another nearby district. Integrated teams consisting of three sub-districts at the cross-boundaries area are required to act in appropriate time-frames without ignoring any issues created by decentralization. Similar to China who adopted a 1-3-7 strategy [38, 39], Indonesia is going to adopt a 1-2-5 strategy, i.e., rapid reporting (day 1), case investigation (day 2) and response in day 5 for all positive cases in all health facilities [40]. To be successful, Indonesia requires a solid, fast response team that is able to do vector control in its cross boundaries areas that will accelerate the target of malaria elimination in Indonesia. Any independent strategic action taken by related districts and provinces in these cross-boundary areas will only waste vital resources and funding without meaningful results.

The finding of this study has been presented to the provincial stakeholders, malaria programmers at Districts and sub-district level related and national authority. All three districts and the two provinces committed to building a partnership to eliminate cross-border malaria. To facilitate the stakeholders in translating this finding into the implementable strategic plan, an operational study “*A participatory approach to address within*-*country cross*-*border Malaria: the case of Menoreh Hills in Java, Indonesia”* has been done sponsored by a donor agency. The outcome of the study was strategic actions developed by the involved parties.

## Conclusions

The use of long-term, intermediate and annual spatiotemporal analyses using ArcGIS and StatsCan in Menoreh Hills lead to the conclusion that primary clusters of malaria in the cross-boundaries areas persistently occurred in Purworejo District. Neglecting this hotspot could potentially delay the malaria elimination in the entire area. The cross-boundary partnership needs to be properly outfitted and reinforced to support malaria elimination in all related districts.

## Appendix

See Table 5.

**Table 5:** Number and villages detected as most likely clusters using the *purely spatial clustering* in cross-boundaries Menoreh Hills Indonesia, 2005–2015

Years	Most likely clusters (villages)		Secondary likely clusters (villages)	
	N	Name of village(s)	N	Name of village(s)
11 years (2005–2015)				
2005–2015	8	Kedunggubah, Kaliharjo, Somongari, Jatirejo, Semagung, Semono, Durensari, Hargorojo	5	Semono, Durensari, Jatirejo, Hargotirto, Kalirejo
2 periods (2005–2010 and 2011–2015)				
2005–2010	24	Hargowilis, Hargotirto, Kalirejo, Hargorojo, Durensari, Semono, Hargomulyo, Dadirejo, Tlogokotes, Somorejo, Hargorejo, Semagung, SokoAgung, Jatirejo, Somongari, Kedunggubah, Jelok, Gunungwangi, Tlogorejo, Hardimulyo, Sudorogo, Ngadirejo, Somowono, Ngaran	11	Ngadirejo, Sudorogo, Hardimulyo, Tlogorejo, Gunung Wangi, Ngaran, Purbowono, Kedunggubah, Jelok, Somowono, Tlogobulu
2011–2015	15	Jelok, Kedung Gubah, Kaligono, Kali Harjo, Hulosobo, Somongari, Jatirejo, Semagung, Soko, Semono, Durensari, Hargo Rojo, Kalirejo, Donorejo, SokoAgung	3	Durensari, Semono, Hargotirto
All years				
2005	9	Hargowilis, Hargotirto, Kalirejo (Kokap), Hargorojo, Durensari, Jatirejo, Semono, Hargomulyo, Donorejo	1	Hargowilis
2006	15	Kaligono, Kedunggubah, Kaliharjo, Tlogoguwo, Ngaran, Donorejo, Purbowono, Jelok, Tlogorejo, Sudorogo, Somongari, Hardimulyo, Tlogobulu, Jatirejo dan Somowono	5	Sudorogo, Tlogobulu, Ngaran, Hardimulyo, Somowono.
2007	6	Hardimulyo, Gunungwangi, Sudorogo, Ngaran, Ngadirejo dan Purbowono.	–	
2008	6	Sudorogo, Ngaran, Hardimulyo, Somowono, Ngadirejo, Purbowono	–	
2009	5	Hardimulyo, Sudorogo, Ngadirejo, Somowono, Piji	–	
2010	10	Sudorogo, Tlogobulu, Hardimulyo, Somowono, Ngadirejo, Tlogorejo, Kaligono, Kemanukan, Semagung, Semono	2	Semono, Semagung
2011	13	Kemanukan, Soko, Semagung, Somongari, Somowono, Kaliharjo, Sokoagung, Hulosobo, Jatirejo, Jelok, Hargorojo, Durensari, Kedunggubah	5	Semono, Durensari, Semagung, Sokoagung, Jatirejo
2012	2	Jelok dan Kedunggubah	9	Jatirejo, Durensari, Somongari, Semono, Hargotirto, Hulosobo, Donorejo, Semagung, Kaliharjo
2013	13	Semagung, Soko, Semono, Clapar, Sokoagung, Somongari, Durensari, Jatirejo, Hargorojo, Kalirejo, Hulosobo, Kaliharjo, Somorejo	2	Sokoagung, Hargorojo
2014	13	Semagung, Soko, Semono, Clapar, Sokoagung, Somongari, Kemanukan, Durensari, Jatirejo, Hargorojo, Kalirejo, Hulosobo dan Kaliharjo	1	Jatirejo
2015	2	Durensari dan Semono	10	Jelok, Tlogorejo, Kaliharjo, Kaligono, Hardimulyo, Ngaran, Hulosobo, Sudorogo, Somongari, Purbowono

*N* number of village (s)
